# Performance of Akaike Information Criterion and Bayesian Information Criterion in Selecting Partition Models and Mixture Models

**DOI:** 10.1093/sysbio/syac081

**Published:** 2022-12-28

**Authors:** Qin Liu, Michael A Charleston, Shane A Richards, Barbara R Holland

**Affiliations:** School of Natural Sciences, University of Tasmania, Hobart, TAS, Australia; School of Natural Sciences, University of Tasmania, Hobart, TAS, Australia; School of Natural Sciences, University of Tasmania, Hobart, TAS, Australia; School of Natural Sciences, University of Tasmania, Hobart, TAS, Australia

## Abstract

In molecular phylogenetics, partition models and mixture models provide different approaches to accommodating heterogeneity in genomic sequencing data. Both types of models generally give a superior fit to data than models that assume the process of sequence evolution is homogeneous across sites and lineages. The Akaike Information Criterion (AIC), an estimator of Kullback–Leibler divergence, and the Bayesian Information Criterion (BIC) are popular tools to select models in phylogenetics. Recent work suggests that AIC should not be used for comparing mixture and partition models. In this work, we clarify that this difficulty is not fully explained by AIC misestimating the Kullback–Leibler divergence. We also investigate the performance of the AIC and BIC at comparing amongst mixture models and amongst partition models. We find that under *nonstandard conditions* (i.e. when some edges have small expected number of changes), AIC underestimates the expected Kullback–Leibler divergence. Under such conditions, AIC preferred the complex mixture models and BIC preferred the simpler mixture models. The mixture models selected by AIC had a better performance in estimating the edge length, while the simpler models selected by BIC performed better in estimating the base frequencies and substitution rate parameters. In contrast, AIC and BIC both prefer simpler partition models over more complex partition models under nonstandard conditions, despite the fact that the more complex partition model was the generating model. We also investigated how mispartitioning (i.e., grouping sites that have not evolved under the same process) affects both the performance of partition models compared with mixture models and the model selection process. We found that as the level of mispartitioning increases, the bias of AIC in estimating the expected Kullback–Leibler divergence remains the same, and the branch lengths and evolutionary parameters estimated by partition models become less accurate. We recommend that researchers are cautious when using AIC and BIC to select among partition and mixture models; other alternatives, such as cross-validation and bootstrapping, should be explored, but may suffer similar limitations [AIC; BIC; mispartitioning; partitioning; partition model; mixture model].

The ongoing development of high-throughput DNA sequencing techniques provides us with large amounts of data for molecular phylogenetic inference. Heterogeneity among homologous sequences in these data may be due to lineage- and site-specific differences in the evolutionary process. This means that simple homogeneous models, which assume equal rates for all sites and lineages, may be inadequate and lead to incorrect phylogenetic inference (Kainer and Lanfear 2015).

Two types of models are commonly used to incorporate heterogeneity in the evolutionary process when analyzing sequencing data: partition models and mixture models. A partition model divides the alignment into subsets of sites (blocks). All sites from the same block are assumed to have evolved under the same evolutionary process, and a different evolutionary model, including a substitution model and a tree, is fit to each block (Lanfear et al. 2012). Depending on the type of the partition models chosen (e.g., an edge-unlinked partition model), the preferred model of evolution could differ in the edge lengths, or in the parameters of the substitution model (Minh et al. 2020). In contrast, a mixture model does not assign sites to different blocks; rather, it fits more than one evolutionary model to each site. Each of these evolutionary models is called a class. A weight factor is placed on each class such that the weights from all classes sum up to one (Lopez et al. 2002). There is evidence to show that both partition models and mixture models can provide a better fit to multigene sequence alignments than homogeneous models (Lartillot and Philippe 2004; Zhou et al. 2007; Le et al. 2008; Darriba and Posada 2015; Baca et al. 2017).

When using partition models, the choice of partition is sometimes based on a priori information about the sequence alignment, for example, by grouping sites based on codon position and/or gene boundaries (Pagel and Meade 2004). More commonly, choosing the partition is treated as part of model selection (Lanfear et al. 2012). The number of possible partitions is typically far too large to explore exhaustively, so algorithmic (heuristic) approaches have been developed to choose between partitioning schemes. For example, *PartitionFinder* and *PartitionFinder2* implement heuristic methods to construct preferred partitioning schemes. Users need to predefine an initial set of blocks. The partitioning scheme is then selected by merging blocks together. The decision to merge blocks is typically based on criteria such as AIC, AICc, and BIC (Lanfear et al. 2012). For data sets with many loci, this may be computationally infeasible, in which case blocks can be merged based on the similarity of estimated model parameters such as base frequencies and rate substitution parameters (Lanfear et al. 2014, 2017).

In recent years, methods have been developed for selecting good partitioning schemes, and investigating the effects of underpartitioning and overpartitioning on phylogenetic analyses (Brandley et al. 2005; Brown and Lemmon 2007; McGuire et al. 2007; Li et al. 2008; Rota and Wahlberg 2012; Leavitt et al. 2013; Kainer and Lanfear 2015; Baca et al. 2017; Lanfear et al. 2017; Tagliacollo and Lanfear 2018; Kim and Sy 2020). The effects of underpartitioning and overpartitioning were examined under different measures, such as tree topologies, edge lengths, edge supports and information criterion (IC) scores including AIC and BIC. Based on different measures, the results differed. One study showed that overpartitioning led to worse IC scores than underpartitioning (Kainer and Lanfear 2015). A few studies showed that the choice of partitioning schemes made no difference to the inferred topologies, edge lengths or both (Brown and Lemmon 2007; Cameron et al. 2012; Kainer and Lanfear 2015). However, other studies showed the opposite results: the choice of partitioning schemes affects tree topologies, edge lengths, and edge support (Ho and Lanfear 2010; Rota and Wahlberg 2012; Leavitt et al. 2013).

Underpartitioning and overpartitioning are not the only issues when partitioning data. Mispartitioning, in which sites are partitioned to the incorrect block, can also lead to inaccurate inferences in both the phylogenies and evolutionary models. However, it is not guaranteed that sites contained in the same subset have evolved under the same evolutionary process in the good partitioning scheme chosen by the traditional approach or even by the algorithmic approach. This is because the latter requires a user predefined partitioning scheme and some sites may have been incorrectly partitioned by the predefined scheme. Brown and Lemmon (2007) and Crotty and Holland (2022) explored the effects of mispartitioning. In their simulations, Brown and Lemmon (2007) used bipartition posterior probabilities as a guideline, and found that the errors produced by mispartitioning were similar to the errors from underpartitioning. Crotty and Holland (2022) found that the accuracy of the topologies and edge lengths inferred by the partition models decreased as the levels of mispartitioning increased. In particular, in their first simulation, if the proportion of incorrectly partitioned sites were 10% or greater, all the topologies estimated by the partition models were different to the generating topologies.

Until recently, most implementations of mixture models have been in a Bayesian framework (Pagel and Meade 2004; Whelan and Halanych 2017), and there are some protein mixture models within a maximum likelihood (ML) framework implemented in IQ-TREE2 (Minh et al. 2020). However, these models have focused on mixtures of substitution processes rather than mixtures of edge weights. Jayaswal et al. (2014) explored models that allowed for heterogeneity across both sites and across lineages. In contrast to most other software, they also allowed for compositional heterogeneity; however, they did not have a software implementation that allowed for optimization of the tree topology. Recently, the GHOST mixture model (General Heterogeneous evolution On a Single Topology) (Crotty et al. 2020) has been implemented in a likelihood framework in IQ-TREE2 (Minh et al. 2020), this allows users to concurrently search model space and tree space, with more than one model fitted to each set of sites (the models are restricted to being stationary and reversible).

Some studies have investigated the performance of partition models and mixture models. In Whelan and Halanych (2017), simulations were performed to assess the performance of partition models and CAT mixture models. The CAT model is a Bayesian mixture model using a Dirichlet process prior to allow for multiple classes of equilibrium frequencies (Lartillot and Philippe 2004). The results showed that the CAT mixture models had poorer performance and higher computational time than the partition models. Therefore, the authors recommended that partition models should be considered first when dealing with heterogeneous data, and that caution should be taken when using the mixture models. However, results in Crotty et al. (2020) suggest that GHOST mixture models are able to detect and recover a subtle evolutionary signal from empirical data. Crotty et al. (2020) analyzed a data set that contained some electric and nonelectric fish and found that the GHOST mixture models recovered a mixture component with edge lengths suggesting convergent evolution of the electric organ in sodium channel genes—because this mixed signal occurred within a single gene, models partitioned based on gene boundaries would not be able to detect it. Crotty and Holland (2022) extended this work further and, focusing solely on the AIC optimality criterion, found that mixture models performed better than partition models, under different levels of mispartitioning settings, in terms of the accuracy of estimated topologies and edge length.

Users of both mixture models and partition models require some way of deciding which, amongst the large variety of possible models, provides the best fit to a particular data set. In both of these cases (partition vs. mixture), the evolutionary processes are assumed to be reversible (and therefore also stationary) over each edge. The most commonly used likelihood-based model selection tool in a phylogenetic context (Posada and Buckley 2004) include the AIC (Akaike 1973 ) and BIC (Schwarz 1978).

AIC incorporates the Kullback–Leibler divergence (KLD) (Akaike 1973), which measures the distances between the true model that generated the data and an approximating model (Kullback and Leibler 1951). In reality, we do not know the true model of observed data, so we cannot use KLD directly to measure the performance of a model. We can, however, estimate the relative expected Kullback–Leibler divergence (rEKL) from the observed data (Burnham and Anderson 2002). AIC does not require knowledge of the underlying true model of the observed data; only the ML of the approximating model, and the complexity of the model defined by the effective number of parameters, *q*.

BIC compares the modified version of the Bayesian posterior probability among candidate models, and selects the model with the best log-likelihood (lnL) after taking into account the penalty factor (i.e., *q* ln(*n*), where *n* is the sample size) (Schwarz 1978). Similarly to AIC, BIC also relies on the ML of a candidate model but BIC differs from AIC in the penalty term calculation (Neath and Cavanaugh 2012).

AIC and BIC are both widely used in model selection, and the best-fit models for the observed data chosen by AIC and BIC are often different (Ota et al. 2000; Posada and Buckley 2004; Dziak et al. 2020). Dziak et al. (2020) compared AIC and BIC in respect to the concepts of sensitivity (i.e., “suggesting enough parameters to accurately model the patterns, processes, or relationships in the data”) and specificity (i.e., “not suggesting nonexistent patterns, processes, or relationships”). The authors showed that AIC emphasizes sensitivity whereas BIC emphasizes specificity (Dziak et al. 2020). Moreover, compared with the AIC, the BIC tends to choose models with fewer parameters and is a “consistent” model selection criterion (Claeskens and Hjort 2008; Dziak et al. 2020). A consistent model selection criterion has a probability of choosing the correct model approaching one, given that the true model is one of the candidate models, as the sample size approaches infinity (Rao and Wu 1989). In other words, AIC allows complex models more often than BIC does, and as the sample size approaches infinity, the probability remains positive that AIC will select a model with more parameters than is necessary.

There is a rich body of literature investigating the use of AIC and BIC in selecting simple homogeneous models in phylogenetics (Posada and Crandall 2001; Posada and Buckley 2004; Sullivan and Joyce 2005; Holder et al. 2010; Luo et al. 2010; Boettiger et al. 2012; Rau and Maugis-Rabusseau 2018). There has been little investigation of the use of AIC and BIC in selecting among partition models and among mixture models. AIC often chooses complex models over simpler models, while BIC inclines to favor the simpler models (Anderson and Burnham 2004; Boettiger et al. 2012; Jhwueng et al. 2014; Dziak et al. 2020). In addition, when the parameters are near the boundaries of the parameter space, both AIC and BIC may perform poorly in model selection (Self and Liang 1987; Ota et al. 2000; Jhwueng et al. 2014; Susko and Roger 2020). The scenario of the parameters being near the boundaries of the parameter space was referred to by Jhwueng et al. (2014) as “nonstandard” conditions. The authors showed that these nonstandard conditions occur when any of the edges in a phylogeny have fewer than 5 expected substitutions per alignment (Jhwueng et al. 2014). Similarly, Susko and Roger (2020) showed that nonstandard conditions also occur when sequences are closely related since the edge lengths for these sequences are near zero. Under these nonstandard conditions, AIC is a negatively biased estimator of the rEKL, and BIC may also be problematic to use under such conditions.

In phylogenetics, the nonstandard conditions are not rare scenarios and can often occur (Felsenstein 2004). Using AIC or BIC under such conditions may lead to choosing a poor model that cannot best explain the data. This issue is likely to be more prevalent for mixture and partition models due to them being more parameter rich. In their simulations, Crotty and Holland (2022) found that, under standard conditions, AIC always preferred partition models over mixture models, when the proportion of allocation of mispartition sites was under 35%, despite the better performance of mixture models in inferring the topologies and edge lengths. In this paper, we extended these results from Crotty and Holland (2022) and investigated the performance of AIC further in nonstandard conditions. Moreover, we also evaluated the performance of BIC in selecting among partition and mixture models under all conditions and compared the performance of AIC and BIC. Crotty and Holland (2022) concluded that the reason for the poor performance of AIC could be that partition models have an “inflated likelihood” compared with mixture models. In our simulations, we examined the performance of AIC from another perspective, that is, whether AIC estimates the rEKL accurately under standard and nonstandard conditions. If not, we wanted to investigate whether this misestimation is another reason to explain preference of AIC for partition models over mixture models even when they are severely mispartitioned.

In this study, we aimed to investigate the use of AIC and BIC to compare partition models and mixture models in standard and nonstandard conditions and we incorporated simulations to address three main questions: (1) Is AIC an unbiased estimator of the rEKL when applied to either partition models or mixture models under either standard or nonstandard conditions, and if so, is there a bias that differs systematically for partition models and mixture models? Does it differ based on the accuracy of the proposed partition? (2) Are models chosen by AIC the same as the ones chosen by BIC? (3) Do the models preferred by AIC and BIC under various levels of mispartitioning generally lead to accurate phylogenetic inference in terms of tree topologies, edge lengths, and substitution model parameters?

## Materials and Methods

### Simulations

To create heterogeneous data, we simulated two multiple sequence alignments (MSAs) under two different simple homogeneous models of DNA evolution. Each model includes a substitution model and an edge-weighted phylogenetic tree (the tree topology was fixed). The differences between these two models were only in the edge lengths and the model parameters. Each MSA contained 8 taxa and 1000 sites. These two MSAs were then concatenated together giving an MSA with 2000 sites. This was equivalent to generating the concatenated MSA under a two-block unlinked edge lengths partition model (P-UEL).

To simulate a situation where the initial choice of blocks does not properly account for the heterogeneity in the concatenated MSA (i.e., mispartitioning), we randomly selected a proportion of 0%, 5%, 10%, 15%, . . ., up to 50% of sites from each block and swapped them. That is, the sites drawn from the first block were placed in the second block, and the sites drawn from the second block were placed in the first block. This process was repeated 100 times for each proportion of mispartitioned sites giving a total of 1100 MSAs.

**Figure 1.**
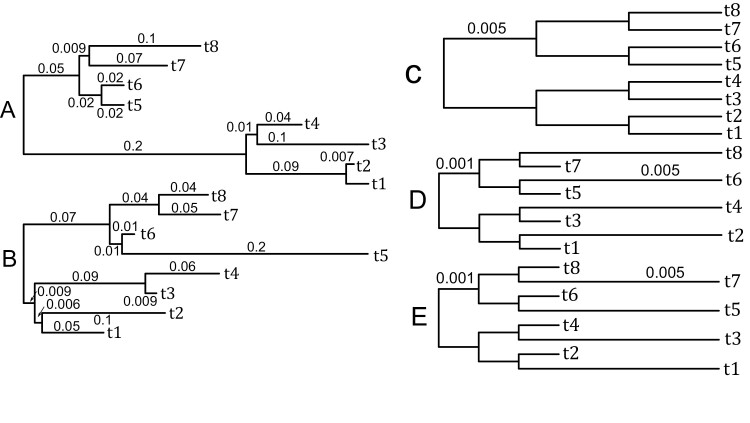
Generating trees used in the simulations. Trees are not to scale. Three pairs of trees {*A*,*B*}, {*C*,*D*}, and {*D*,*E*} are the generating trees for standard, mild, and extreme simulations, respectively. The edge lengths for tree C and the longer edge lengths for trees D and E are the same with a length of 0.005. The shorter edges of trees D and E are 0.001.

Three sets of simulations were created under standard, mildly nonstandard and extremely nonstandard conditions (denoted as standard, mild, and extreme). The generating trees for all simulations had the same topologies but different edge lengths (Figure 1). We used the same tree topology as that in Jhwueng et al. (2014). In the standard simulation, all edge lengths were randomly drawn from an exponential distribution with a mean of 0.06, such that the expected number of substitutions per block for each edge in both trees was greater than 5. (Where the expected number of substitutions is the product of the edge length and the number of sites in the block.) For the mild simulation, all edges in the tree have edge lengths of 0.005, that is, the expected number of simulated substitutions per block for each edge was 5 (as in Jhwueng et al. (2014)). The second generating tree in the mild simulation had edge lengths of either 0.005 or 0.001. That is, the expected number of substitutions per block for each edge was either 5 or 1. The second tree had the potential to generate a long branch attraction (LBA) artifact as the long edges do not form a monophyletic group. For the extreme simulation, both generating trees had edge lengths of either 0.005 or 0.001 and both trees had potential to cause LBA problems.

The parameters of the generating substitution models are the same for all three simulations (Table 1). The method of creating the substitution rate parameters and base frequencies was the same as in Crotty et al. (2020). The G↔T rate is fixed at 1, and the other 5 transition rates were drawn randomly from a uniform distribution in [0.5,5]. To generate random base frequencies that were not too extreme, we randomly drew four uniform random numbers on [0,1], and normalized them to sum to 0.6, then we added 0.1 to each value.

**Table 1:** Parameters of the generating two-block unlinked edge lengths partition models (P-UEL) for three simulations

Model	Rate matrix	Base frequencies
GTR (block 1)	$Q_{1}=\left(1.56,3.20,4.02,1.32,0.90,1\right)$	$\pi_{1}=\left(0.28,0.34,0.19,0.19\right)$
GTR (block 2)	$Q_{2}=\left(1.17,2.41,1.79,2.65,2.99,1\right)$	$\pi_{2}=\left(0.17,0.30,0.37,0.16\right)$

We fitted two partition models and two mixture models to each MSA. These models are a two-block P-UEL partition model, a two-block linked edge lengths partition model (P-LEL), a two-class linked GTR parameters GHOST mixture model (M-LGP) and a two-class unlinked GTR parameters GHOST mixture model (M-UGP). A detailed explanation of these models is shown in the Models and Performance Measures section.

The data were generated under Seq-Gen-1.3.4 (Rambaut and Grass 1997) and model fitting and tree search were performed in IQ-TREE2 (Minh et al. 2020). Tree search was the IQ-TREE2 default tree search that combines hill-climbing algorithms and a “stochastic perturbation method,” with 100 parsimony trees and BIONJ trees as the starting trees (Minh et al. 2020). All results were analyzed using the R package phangorn (version 3.6.2) (Schliep 2011; R Core Team 2019). Custom bash scripts are used to extract relevant parts of the results from IQ-TREE2 for processing in R. The data, R codes and bash scripts, are available in the Supplementary Material available on Dryad (doi:10.5061/dryad.1jwstqjwj).

### Models and Performance Measures

#### Partition models and mixture models

Pagel and Meade (2004) derived an expression for a generalized mixture model and showed that both partition and mixture models are special cases of this generalized mixture model. This expression is Equation (5.1) in Gascuel (2005) and it assumes that only one specific tree is used for the generalized mixture model. However, heterogeneities may also occur along all edges (Crotty et al. 2020), so we modified this equation assuming heterogeneities exist across both sites and edges, and the lnL of an alignment under a generalized mixture model is


$$\begin{array}{l} \begin{array}{l} ln\left(L(D|M_{1},M_{2},\ldots ,M_{K},T_{1},T_{2},\ldots ,T_{K})\right)\\ =ln\left(\prod_{i}\sum_{k}\alpha_{ik}L(D_{i}|M_{k},T_{k})\right)\\ =\sum_{i}ln\left(\sum_{k}\alpha_{ik}L(D_{i}|M_{k},T_{k})\right), \end{array} \end{array}$$ (1)

where $L\left(D\;|\;M_{1},M_{2},\ldots ,M_{K},T_{1},T_{2},\ldots ,T_{K}\right)$ is the likelihood of an alignment under the mixture model, *K* is the total number of classes, *M*_*k*_ and *T*_*k*_ are the substitution model and the tree of the kth class, respectively, *D* is an alignment, *D*_*i*_ is site *i* in *D*, $\alpha_{ik}$ is the weight for the *k*th class for site *i* and $\sum_{k}\alpha_{ik}=1$.

In Equation 1, if the weight of each class is held constant across all sites, then we obtain the lnL function of an alignment for a mixture model (Gascuel 2005). Alternatively, if the weight of one particular class is set to 1, and the weights of the rest of the classes are set to 0 for some sites, then we get the lnL function for a partition model. In other words, the structure of a partition model is to fit one evolutionary model to a subset of sites, and different subsets of sites are presumed to have evolved under different evolutionary processes. This partition structure is assumed to be known, and the equation of the partition model can be derived from Equation 1 as follows.


$$\begin{array}{ll} & ln\left(L(D|M_{1},M_{2},\ldots ,M_{K},T_{1},T_{2},\ldots ,T_{K})\right)\\ & =\sum_{k=1}^{K}\sum_{i\in S_{k}}ln\left(w_{ik}L(D_{i}|M_{k},T_{k})\right), \end{array}$$ (2)

where *K* is the total number of partitions, and if site *i* is in the set *S*_*k*_ = {*i*: *w*_*ik*_ = 1}, otherwise *w*_*ik*_ = 0.

The four models we used in the simulations were P-LEL, P-UEL, M-UGP, and M-LGP. The first two models are both two-block partition models and the last two models are both two-class GHOST mixture models. In a P-LEL model, all blocks of sites share the same set of edge lengths but have their own substitution rate parameters. The partitioning schemes used for both fitted partition models were the same, treating the first 1000 sites as a block and the second 1000 sites as another. For the groups of MSAs with different proportions of mispartitioned sites, this was equivalent to fitting the partition models with an incorrect partitioning scheme.

In the two fitted GHOST mixture models, each class always has its own set of edge lengths, but may or may not share the same GTR substitution model. In an M-LGP model, each class shares the same GTR substitutional model, while in an M-UGP model, each class has its own GTR substitutional model.

Using the same notation as in Equations 1 and 2, we can derive the lnL for these four partition and mixture models, and these lnL expressions are special cases of Equation 1:


$$\begin{array}{l} \begin{array}{lll} & ln\;L_{P-LEL}= & ln\left(P(D|M_{1},M_{2},T)\right)\\ & = & \sum_{i=1}^{1000}ln\left(L(D_{i}|M_{1},T)\right)+\sum_{i=1001}^{2000}ln\left(L(D_{i}|M_{2},T)\right) \end{array} \end{array}$$ (3)

$$\begin{array}{l} \begin{array}{lll} & ln\;L_{P-UEL}= & ln\left(P(D|M_{1},M_{2},T_{1},T_{2})\right)\\ & = & \sum_{i=1}^{1000}ln\left(L(D_{i}|M_{1},T_{1})\right)+\sum_{i=1001}^{2000}ln\left(L(D_{i}|M_{2},T_{2})\right) \end{array} \end{array}$$ (4)

$$\begin{array}{l} \begin{array}{lll} & ln\;L_{M-UGP}= & ln\left(P(D|M_{1},M_{2},T_{1},T_{2})\right)\\ & = & \sum_{i=1}^{2000}ln\left(\alpha_{1}L(D_{i}|M_{1},T_{1})+\alpha_{2}L(D_{i}|M_{2},T_{2})\right) \end{array} \end{array}$$ (5)

$$\begin{array}{l} \begin{array}{lll} & ln\;L_{M-LGP}= & ln\left(P(D|M,T_{1},T_{2})\right)\\ & = & \sum_{i=1}^{2000}ln\left(\alpha_{1}L(D_{i}|M,T_{1})+\alpha_{2}L(D_{i}|M,T_{2})\right) \end{array} \end{array}$$ (6)

The details of the fitted models and the number of the free parameters are shown in Tables 2 and 3.

**Table 2:** Details of the fitted models

Model name	Full name	Edges	Substitution model	Number of parameters
				GTR+GTR
P-LEL	2-block *L*inked *E*dge *L*engths			
	partition model	linked	unlinked	30
P-UEL	2-block *U*nlinked *E*dge *L*engths			
	partition model	unlinked	unlinked	42
M-UGP	2-class *U*nlinked *G*TR *P*arameters			
	GHOST mixture model	unlinked	unlinked	43
M-LGP	2-class *L*inked *G*TR *P*arameters			
	GHOST mixture model	unlinked	linked	35

The last column shows the number of parameters needed to estimate the model. The partitioning scheme used for both partition models was the same: the first 1000 sites as a block and the second 1000 sites as another block.

**Table 3:** Numbers of the free parameters for the fitted models

	Substitution model	Edge lengths		
Model name	GTR1 + GTR2	Tree1 + Tree2	Class weights	Total
	(or one GTR for M-LGP)	(or one tree for P-LEL)		
P-LEL	8 + 9	13	NA	30
P-UEL	8 + 8	13 + 13	NA	42
M-UGP	8 + 8	13 + 13	1	43
				
M-LGP	8	13 + 13	1	35

For the P-LEL model, one GTR model has 8 free parameters, but another GTR model has 9. This is because the P-LEL model shares the same edge lengths, and we cannot distinguish the times and the substitutional rates. Therefore, for the P-LEL model, if the rate of G→T in the substitution rate parameters of a GTR model is set to one, then the rate of G→T in the other GTR model need not be set to 1.

#### Relative expected Kullback–Leibler divergence, AIC, bias of AIC, and BIC

rEKL is the measure we wanted to estimate in this study and is derived from the KL divergence between the true distribution and an approximating model (Burnham and Anderson 2002).

Let X and Y denote two independent alignments both generated by the same model, i.e., drawn from the same multinomial distribution over site patterns, then the KL divergence between the generating model and a candidate model using X for estimation is (Burnham and Anderson 2002):


$$\begin{array}{l} KL(X)=\sum_{Y\in \Omega }p(Y)ln\left(\frac{p(Y)}{\hat{p}(Y|{\hat{\theta }}_{X}))}\right), \end{array}$$ (7)

where Ω is the space of all possible alignments, ${\hat{\theta }}_{X}$ is the ML estimate (MLE) of the model parameters, and $ln\;[\;\hat{p}(Y|{\hat{\theta }}_{X})\;]\;$ is the ln*L* of Y given the MLE of *X*.

Equation 7 is the KL divergence between the truth and a candidate model and it is difficult to compute in reality since the truth is unknown. Instead, we can obtain the expectation of Equation 7 by taking an average over all values of X (Burnham and Anderson 2002). The expected KL divergence (EKL) is


$$\begin{array}{ll} EKL & =\sum_{X\in \Omega }p^{*}(X)KL(X)\\ & =\sum_{X\in \Omega }p^{*}(X)\sum_{Y\in \Omega }p^{*}(Y)(lnp^{*}(Y)-ln\hat{p}(Y|{\hat{\theta }}_{X}))\\ & =\sum_{X\in \Omega }p^{*}(X)\sum_{Y\in \Omega }p^{*}(Y)lnp^{*}(Y)\\ & \;-\sum_{X\in \Omega }p^{*}(X)\sum_{Y\in \Omega }p^{*}(Y)ln\hat{p}(Y|{\hat{\theta }}_{X}), \end{array}$$ (8)

where p*(*X*) and p*(*Y*) are the true probabilities of alignments *X* and *Y*, and $\hat{p}\left(Y\;|\;{\hat{\theta }}_{X}\right)$ is probability of *Y* given the MLE of *X*. The first term in Equation 7 does not depend on the candidate model and can be ignored. The second term in Equation 8 multiplied by 2 is the rEKL used in this study. This is the measure that we wanted to estimate and compare to AIC in the simulations.


$$\begin{array}{l} rEKL=-2\sum_{X\in \Omega }p^{*}(X)\sum_{Y\in \Omega }p^{*}(Y)ln\hat{p}(Y|{\hat{\theta }}_{X}). \end{array}$$ (9)

The term $\sum_{Y\in \Omega }p^{*}(Y)\;ln\;\hat{p}(Y\;|\;{\hat{\theta }}_{X})$ in Equation 9 can be re-expressed as a sum over all possible site patterns rather than a sum over alignments, as discussed in Jhwueng et al. (2014):


$$\begin{array}{l} rEKL=-2\sum_{X\in \Omega }p^{*}(X)\sum_{h=1}^{4^{N}}np^{*}(h)\;ln\;\hat{p}(h|{\hat{\theta }}_{X}), \end{array}$$ (10a)

$$\begin{array}{l} =-2\sum_{h=1}^{4^{N}}np^{*}(h)\sum_{X\in \Omega }p^{*}(X)ln\;\hat{p}(h|{\hat{\theta }}_{X}), \end{array}$$ (10b)

where p*(*h*) is the true probability of the site pattern *h*, $\hat{p}(h\;|\;{\hat{\theta }}_{X})$ is the estimated probability of the site pattern *h* under the MLE of the candidate model, and *N* and *n* are the total number of taxa and the total number of sites in an alignment, respectively. It is worth noting that Susko and Roger (2020) used the expected predictive log-likelihood (EPLnL) as an equivalent target of AIC approximation, and EPLnL=−(1/2)(rEKL).

As in Jhwueng et al. (2014), equation 10b can be estimated using simulated data sets:


$$\begin{array}{l} rEKL\approx -2\sum_{h=1}^{4^{N}}np^{*}(h)\left(\frac{1}{c}\sum_{l=1}^{c}ln{\hat{p}}^{l}(h|{\hat{\theta }}_{X})\right), \end{array}$$ (11)

where *c* is the total number of simulated MSAs, and ${\hat{p}}^{l}\left(h\;|\;{\hat{\theta }}_{X}\right)$ is the estimated probability of the site pattern *h* under an approximating model when fitted to the *l*th simulated alignment.

We modified Equation 11 to calculate the rEKL for the two-block partition models (Equation 12) and two-class mixture models (Equation 13) with different numbers of mispartitioned sites. This gives


$$\begin{array}{l} \begin{array}{lll} & rEKL_{P}\approx & -2\sum_{h=1}^{4^{N}}\left(\sum_{j=1}^{2}\left(\sum_{i\in S_{j}}w_{ij}p^{*j}(h)\right)\right)\sum_{k=1}^{2}\left(\frac{1}{c}\sum_{l=1}^{c}ln({\hat{p}}^{lk}(h))\right)\\ & & \;\;\;\;\;=-2\{\sum_{h=1}^{4^{N}}\left(\left(\sum_{i\in S_{1}}p^{*1}(h)+\sum_{i\in S_{2}}p^{*2}(h)\right)\frac{1}{c}\sum_{l=1}^{c}ln({\hat{p}}^{l1}(h))\right)\\ & & \;+\sum_{h=1}^{4^{N}}\left(\left(\sum_{i\in S_{1}}p^{*1}(h)+\sum_{i\in S_{2}}p^{*2}(h)\right)\frac{1}{c}\sum_{l=1}^{c}ln({\hat{p}}^{l2}(h))\right)\}, \end{array} \end{array}$$ (12)

where *h* indexes the site patterns, *k* indexes the partitions to which the sites have been assigned, *j* indexes components of the generating process (the truth), the set *S*_*j*_ indexes the true partition structure (*S*_*j*_ = {*i*: *w*_*ij*_ = 1}, otherwise *w*_*ij*_ = 0), p*^J^(*h*) is the true probability of site pattern *h* for the jth partition, and ${\hat{p}}^{lk}\left(h\right)$ is the estimated probability of site pattern *h* for the *k*th partition when fitted to the simulated alignment *l*.


$$\begin{array}{l} \begin{array}{ll} & rEKL_{M}\approx -2\sum_{h=1}^{4^{N}}\left(\sum_{i=1}^{n}\left(\sum_{j=1}^{2}\left(\alpha_{j}p^{*j}(h)\right)\right)\right)\left(\frac{1}{c}\sum_{l=1}^{c}ln(\sum_{k=1}^{2}{\hat{\alpha }}_{k}^{l}{\hat{p}}^{lk}(h))\right)\\ & \;\;\;=-2\sum_{h=1}^{4^{N}}\left(n\left(\alpha_{1}p^{*1}(h)+\alpha_{2}p^{*2}(h)\right)\left(\frac{1}{c}\sum_{l=1}^{c}(ln({\hat{\alpha }}_{1}^{l}{\hat{p}}^{l1}(h)+{\hat{\alpha }}_{2}^{l}{\hat{p}}^{l2}(h)))\right)\right), \end{array} \end{array}$$ (13)

where p*^j^(*h*) is the true probability of site pattern *h* for the *j*th class, ${\hat{p}}^{lk}\left(h\right)$ is the estimated probability of site pattern *h* for the *k*th estimated class when fitted to the simulated alignment *l*, $\alpha_{j}$ is the true class weight and ${\hat{\alpha }}_{k}^{l}$ is the estimated class weight for the simulated alignment *l*, and $\alpha_{1}+\alpha_{2}={\hat{\alpha }}_{1}^{l}+{\hat{\alpha }}_{2}^{l}=1$.

The values of AIC, BIC, and the bias in the AIC estimate of rEKL are also recorded. The equation of AIC for the model of interest is $AIC=-2\times ln\hat{L}+2q$, where $ln\hat{L}$ is the ML of the data given the model. The bias of AIC is calculated following a similar approach to that given in Jhwueng et al. (2014). Bias was defined as: Bias(AIC) = rEKL − E(AIC), where E(AIC) is the mean AIC score for a set of simulated alignments.

We used a different version of BIC for partition models. The BIC for mixture models is the same as the conventional one: $BIC=-2\times ln\hat{L}+qln\left(n\right)$. For the partition models, the BIC derived in Susko and Roger (2020):


$$\begin{array}{l} BIC=-2\times ln\hat{L}+\left(\sum_{k=1}^{K}q_{k}ln(n_{k})\right)+q_{c}ln(n), \end{array}$$ (14)

where *n*_*k*_ is the number of sites in the *k*th partition, *q*_*k*_ is the number of parameters unique to the *k*th partition, *q*_*c*_ is the number of parameters common to all partitions, and $q=\sum_{k}q_{k}+q_{c}$ is the total number of the free parameters.

#### Branch score

The branch score, developed by Kuhner and Felsenstein (1994), is a measure of distance between two trees that accounts for differences in edge length as well as topology. For the P-LEL model, we compared the shared set of the inferred edge lengths to both of the generating trees, recorded the branch scores and took the average of the two branch scores. For the P-UEL model, we compared the inferred edge lengths from the first and second blocks of the MSAs to the first and the second generating trees, respectively, and took the average of the two branch scores. For the M-UGP and M-LGP mixture models, we calculated the branch scores in two ways: comparing the Class 1 edge weights to the first generating tree and Class 2 edge weights to the second generating tree, and visa versa. Then we calculated the weighted average branch scores for each option, and we took the minimum of these two options.

#### Estimated base frequencies and substitution rate parameters

We performed element-wise comparisons between the estimated base frequencies and the generating base frequencies, and between the estimated substitution rate parameters and the generating substitution rate parameters. For the partition models, we compared the base frequencies and substitution rate parameters estimated from the first and second blocks of the MSA to the generating base frequencies and substitution rate parameters from the first and second blocks of MSA, respectively. For the mixture models, we compared the estimated base frequencies and substitution rate parameters from the classes with the two sets of generating parameters based on the allocation where the edge lengths match best.

## Results

### Bias of AIC

**Figure 2.**
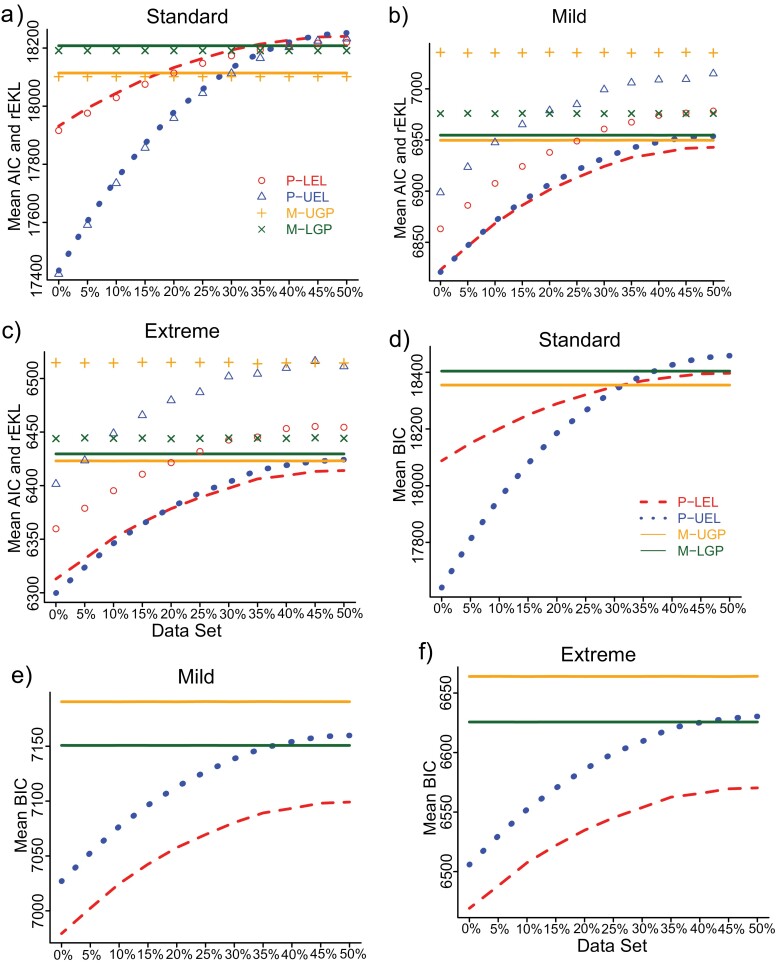
Mean AIC (left), the rEKL (left), and mean BIC (right) results for three simulations. The label on the *x*-axis represents the groups of MSAs with different proportions of incorrectly partitioned sites. The dots represent the rEKL values and the lines the mean AIC and BIC.

Under standard conditions, AIC was an unbiased estimator of the rEKL. The models with the smallest mean AIC also had the smallest rEKL values, that is, both the AIC and rEKL favored the same models (Fig. 2a). For MSAs with 0% to 25% of mispartitioning sites, both the rEKL and AIC preferred the P-UEL partition models for these groups of MSAs. As the incorrectly partitioned sites increased, for MSAs with 30% to 50% of mispartitioning sites, both rEKL and AIC chose the M-UPG mixture models most of the time.

Under nonstandard conditions, AIC was a biased estimator of rEKL (Fig. 2b,c), and this result is consistent with those in Jhwueng et al. (2014). The mean Bias(AIC) is greater for more complex models (i.e., P-UEL and M-UGP) under nonstandard conditions (Table 4). The differences in the mean Bias(AIC) between a partition model and a mixture model are similar under mild and extreme simulations. In the mild simulations, the mean Bias(AIC) of both partition models is greater than the M-LGP mixture model, and is smaller than the M-UPG mixture models. In the extreme simulations, the mean bias of the M-LGP model is still the smallest, but the mean Bias(AIC) of both partition models are not always smaller than the M-UGP mixture model (Table 4).

**Table 4:** Bias (AIC) in estimating the relative expected Kullback–Leibler divergence (rEKL) values for 0% and 50% groups of MSAs for 3 simulations. Bias(AIC) = rEKL − E(AIC). The Bias(AIC) were the same for the GHOST mixture models across all groups of MSAs, so only one value is shown for the mixture models. The Bias(AIC) for the partition models were very similar to each other, so only the Bias(AIC) from 0% and 50% groups are shown in the table

Simulation	Group	Mean bias (AIC)			
		P-LEL	P-UEL	M-UGP	M-LGP
Standard	0 %	15.4	11.4	12.4	16.8
	50 %	20.5	20.0		
Mild	0 %	−40.1	−77.8	−86.0	−21.2
	50 %	−35.5	−61.2		
Extreme	0 %	−46.8	−101.8	−91.7	−14.3
	50 %	−40.4	−87.0		

### Bayesian Information Criterion

Under standard simulations, the result is the same as the result from AIC. That is, BIC preferred the P-UEL partition models from MSAs with 0% to 25% mispartitioning sites, and for the rest of the groups, BIC chose the M-UGP mixture models (Fig. 2d). Under mild and extreme conditions, BIC always chose the simpler P-LEL partition models over the more complex partition models (P-UEL) (Fig. 2e,f). This result is different to the result from AIC. That is, under mild and extreme simulations, AIC chose the complex P-UEL partition model when the MSAs contained none or a small amount (up to 15%) of mispartitioning sites, and chose the simpler P-LEL partition models for the rest of the MSAs (Fig. 2b,c). In summary, under mild and extreme conditions, BIC always selected the simpler P-LEL partition models while AIC preferred both partition models depending on the amount of mispartitioning sites in the data.

### Performance of Models Chosen by AIC and BIC Under Various Levels of Mispartitioning

**Figure 3.**
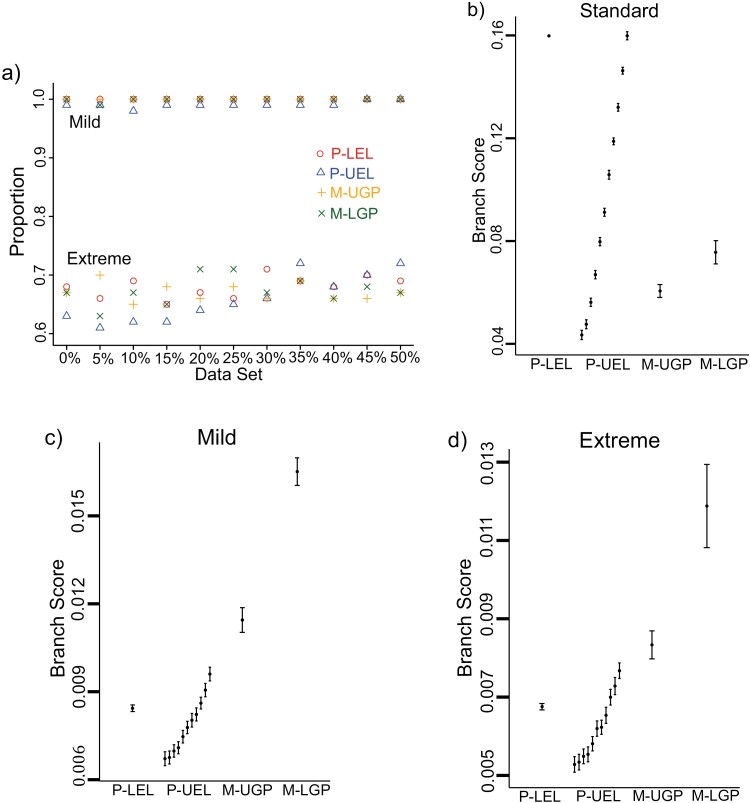
Proportion of the MSAs with the inferred topology the same as the generating topology and branch scores of the inferred trees to the generating tree 1 for three simulations. (a) Proportion of the time the trees inferred by each model recovered the correct generating tree topologies for different group of MSAs in mild and extreme simulations. The result is not shown for standard simulation, since the inferred trees from four models in this simulation all correctly recovered the generating tree topologies. (b)–(d) The mean branch scores for three simulations. Vertical bars indicate ±2 standard errors of the mean branch scores. The 11 bars for the P-UEL models, from left to right, represent the branch scores, for 0% to 50% groups of MSAs, respectively. Only one bar is shown for the P-LEL and the mixture models since mispartitioning has little effect (less than 1% difference in the mean branch scores) on the performance of these models in estimating the generating edge lengths.

In the standard and mild simulations, models chosen by AIC and BIC all recovered the true topologies very well, 100% and nearly 100% of the time for all MSAs, respectively (Fig. 3a). In the extreme simulation, the proportion of the time the trees inferred by the models successfully recovered the generating topologies was in the range of [61%, 72%] (Fig. 3a), and these differences in accuracy between models were not statistically significant.

Mispartitioning had no impact on the accuracy of the topologies inferred by the partition and the mixture models (Fig. 3a). The proportion of the time that a model recovered the generating topology remained the same as the incorrectly partitioned sites increased. This probably was due to the two generating trees having the same topology in the simulations.

Under standard conditions, mixture models had a better (lower) branch score than partition models when the proportion of incorrectly partitioned sites was 15% or greater (Fig. 3b). Under nonstandard conditions, partition models performed better than mixture models in estimating the generating edge lengths (Fig. 3c,d). Mispartitioning seemed to only affect the inferred edge lengths for the P-UEL partition model but not for the other partition model. As the mispartitioned sites increased, the accuracy of the branch scores decreased for the P-UEL partition model.

**Figure 4.**
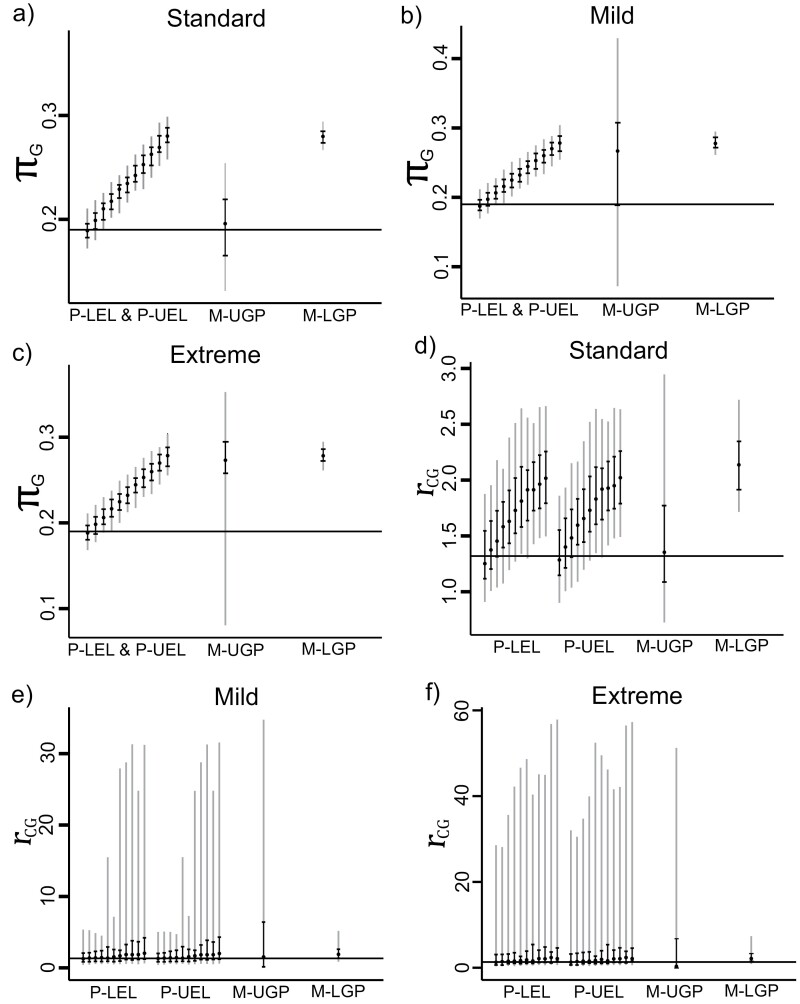
Estimated base frequency $\pi_{G}$ (left) and evolutionary rate $r_{CG}$ (right) of block one for three simulations. Each vertical bar indicates the (25^th^, 75^th^) percentiles (black) and the (5^th^, 95^th^) percentiles (grey) of the sampling distribution. The dot on each vertical bar shows the median estimated $\pi_{G}$ or $r_{CG}$. The horizontal solid black lines are the generating $\pi_{G}$ or $r_{CG}$ from block one of the sequences. The estimated base frequencies of the two partition models were the same. The 11 bars for partition models, from left to right, represent the estimated $\pi_{G}$ or $r_{CG}$, for 0% to 50% groups of MSAs, respectively. Only one bar is shown for each mixture model since mispartitioning does not affect the performance of mixture models in estimating the generating parameters.

We created element-wise comparisons to assess the accuracy of the estimated base frequencies and substitution rate parameters. The accuracy of each element in the estimated base frequencies and substitution rate parameters was similar, so we only showed the distribution of the inferred $\pi_{G}$ from the base frequencies and the inferred *r*_*CG*_ from the substitution rate parameters from block one of the partition model and one class from the mixture model (Fig. 4). The combination of the comparison for the mixture model (M-UGP) was determined based on the allocation of the best mean branch scores. Figure 4 shows the median, the 50% and 90% quantiles of the sampling distribution for the estimated $\pi_{G}$ and $r_{CG}$ for block one of the partition model and one class from the mixture model.

Under standard conditions, based on the 50% quantile of the sampling distribution, one mixture model (M-UGP) performed better at estimating the base frequencies than the partition models when the mispartitioning level was 20% and above (Fig. 4a). Under nonstandard conditions, the 50% quantile of the sampling distribution shows that the mixture models performed equally well at estimating the base frequencies as the partition models when the mispartitioning was severe (40% and above) (Fig. 4b,c).

In general, the accuracy of the estimated substitution rate parameters for the mixture models is similar to the ones from the partition models (Fig. 4d–f). Under standard conditions, based on the 50% quantile of the sampling distribution, one mixture model (M-UGP) had a better accuracy at estimating the generating substitution rate parameters (Fig. 4d). Under nonstandard conditions, the sampling distributions for the two partition models and one mixture model (M-UGP) were heavily right skewed. The 50% quantiles of the partition models and mixture models all captured the generating substitution rate parameters under nonstandard conditions (Fig. 4e,f).

In summary, under nonstandard conditions, compared with the two partition models, BIC chose the simpler partition models (P-LEL) across all levels of mispartitioning. The AIC preferred the simpler partition models over the more complex partition models when there are more than 15% of sites are mispartitioned in the MSAs. Between the two mixture models, BIC preferred the simpler mixture models across all data sets. However, AIC chose the complex mixture models over the simpler ones for all the simulated data. Under nonstandard conditions, the simpler partition models chosen by AIC and BIC performed better than the complex partition models in estimating the edge lengths (Fig. 3c,d). However, the edge lengths estimated by the complex mixture models chosen by AIC were more accurate than the ones of the simple mixture model chosen by BIC.

## Discussion

AIC and BIC are two popular tools to use when analyzing simple homogeneous models in phylogenetics. There is limited work on the performance of AIC and BIC in selecting among partition models and mixture models. Here we have shown that AIC underestimates the rEKL under nonstandard conditions. This result is consistent with Jhwueng et al. (2014). In general, under nonstandard conditions, the partition models selected by both AIC and BIC were the same when the mispartitioning level was 15% or above in the MSAs. However, when comparing the two mixture models, AIC chose the complex mixture models over the simpler ones, while BIC always preferred the simpler mixture models.

The inconsistent bias of AIC in estimating the rEKL among models, under different nonstandard conditions, suggests an incorrect calculation of the effective number of parameters. Under nonstandard conditions, in which the parameters are near the boundary of the parameter space, the effective number of parameters should not be calculated using such an approach (Moody 1992; Spiegelhalter et al. 2002). In a linear model setting, Burnham and Anderson (2002) derived an equation (Equation 7.53) to calculate the effective number of parameters for models with singularities and boundary problems. This effective number of parameters is difficult to obtain by using this equation in a phylogenetic setting, since it requires the calculation of the covariance of the random observations, which is challenging to compute for sequence alignments. In addition, the larger the number of parameters a model has, the more difficult the estimation is. This may also contribute to the inconsistency of the bias of AIC in estimating rEKL among models.

This study verified the results from Crotty and Holland (2022) that partition models and mixture models are not comparable using AIC, not only under standard conditions but also under nonstandard conditions. This result cannot be explained by the bias of AIC in estimating the rEKL. The difference in the likelihood functions of a partition model and a mixture model could be a possible reason for the AIC and BIC choosing partition models over mixture models. A partition model fits the best model to each block of sites, and the log-likelihood is obtained by summing up all the log-likelihood from each individual model from each block (Lanfear et al. 2017). A mixture model fits multiple models to each site, and the log-likelihood is obtained by summing all the weighted averages of the log-likelihood from these models across all sites (Pagel and Meade 2004). This may lead to a partition model having a higher (better) maximum log-likelihood than a mixture model, in some situations as discussed in Crotty and Holland (2022). Similar to AIC, BIC, and rEKL also have the same issue that makes comparing between mixture and partition models infeasible.

Our simulations showed that, under nonstandard conditions, AIC and BIC chose the same simpler partition models (P-LEL) when more than 15% of sites are mispartitioned. This result is interesting in terms of the bias variance trade-off. BIC favors an underfitting model which has a high bias and a low variance (specificity) and AIC prefers an overfitting model which has a low bias and a high variance (sensitivity) (Altman and Bland 1994; Hastie et al. 2009; Dziak et al. 2020). However, in our simulations under nonstandard conditions, both AIC and BIC chose the same model. This could be because the sample size may not be large enough (*n* = 2000). The simpler partition models may be the best optimized solutions for both AIC and BIC despite the fact that AIC and BIC choose models that give different aspects in terms of the bias variance trade-off. In these circumstances, AIC and BIC may not fail, a simpler partition model may just explain the true process that generated the MSAs better than the other models for a smaller *n*. With a larger *n*, the choice of AIC and BIC may differ. This result also implies that, under nonstandard conditions, AIC and BIC may favor partition models with an underpartitioned scheme over partition models with an overpartitioned scheme.

There is a slight difference in the results between BIC and AIC under nonstandard conditions when there was none (i.e., 0%) or some small amounts (i.e., up to 15%) of mispartitioning sites in the MSAs. That is, BIC always preferred the simpler partition model (i.e., P-LEL model), while AIC selected the generating model (P-UEL). This result is consistent with the assertion made in Dziak et al. (2020): BIC often chooses a simpler model while AIC a more complex model. Interestingly, rEKL also selected the simpler partition models in this situation. It is worth noting that, for the 0% MSAs, the P-UEL partition model was the generating model, and AIC chose this model but rEKL and BIC preferred the P-LEL model. The result from rEKL indicates that the simpler P-LEL model has the smallest KLD from the true model, and this model may explain the variation of the data better than the generating model, even under the incorrect simplifying assumptions. In this case, perhaps none of the choices was wrong, the inconsistency of the results reflects different aspects of the model performance that AIC and BIC focus on.

Furthermore, our simulations showed that when dealing with heterogeneous data, both partition models and mixture models performed well in estimating different aspects of phylogenetic inference, and the computational time for fitting the GHOST mixture models to the data is similar to the time for the partition models in IQ-TREE2. This result is consistent with Crotty et al. (2020), but inconsistent with Whelan and Halanych (2017).

Overall, AIC and BIC may not be appropriate to use in selecting among partition models and mixture models under nonstandard conditions. Nonstandard conditions can occur in many situations: for example, when an MSA has some closely related species, or when partitioning data into small subsets, nonstandard conditions may occur. As Susko and Roger (2020) pointed out, if an MSA contains some closely related species, the tree inferred from the MSA contains some edges with a near zero edge length.

Caution should be taken if using AIC and BIC to select among partition models and mixture models when dealing with these types of MSAs. We recommend that when fitting a partition model and a mixture model to the data, the inferred phylogenetic trees should be inspected prior to final model selection. Furthermore, when partitioning data into a large number of subsets, such that each subset has a very small sequence length, AIC and BIC may become problematic to use. This is because if the sequence length is very small, then the expected number of substitutions per alignment may be less than 5, creating a nonstandard condition. We recommend that when fitting a partition model to the data, the sequence length of the partitions should be examined. We also recommend that alternative estimators of the rEKL are worth investigating and considering in selecting between partition models and mixture models. These estimators include cross-validation scores proposed in Susko and Roger (2020), and bootstrapping scores derived in Jhwueng et al. (2014). However, it is possible that these approaches will also not provide a “magic bullet” by which mixture models and partition models can be fairly compared.

## Supplementary Material

Data available from the Dryad Digital Repository: http://dx.doi.org/10.5061/dryad.1jwstqjwj

## References

[CIT0001] Akaike H. 1973. Information theory as an extension of the maximum likelihood principle. Second International Symposium on Infromation Theory. Budapest: Czaki, Akademiai Kiado. p. 276–281.

[CIT0002] Altman D.G. , Bland J.M. 1994. Diagnostic tests. 1: sensitivity and specificity. BMJ 308:1552. 8019315 10.1136/bmj.308.6943.1552 PMC2540489

[CIT0003] Anderson D. , Burnham K. 2004. Model selection and multi-model inference. 2nd ed. New York: Springer. p. 10.

[CIT0004] Baca S.M. , Toussaint E.F., Miller K.B., Short A.E. 2017. Molecular phylogeny of the aquatic beetle family Noteridae (Coleoptera: Adephaga) with an emphasis on data partitioning strategies. Mol. Phylogenet. Evol. 107:282–292. 27789326 10.1016/j.ympev.2016.10.016

[CIT0005] Boettiger C. , Coop G., Ralph P. 2012. Is your phylogeny informative? Measuring the power of comparative methods. Evol. Int. J. Org Evol. 66:2240–2251. 10.1111/j.1558-5646.2011.01574.x PMC3448289 22759299

[CIT0006] Brandley M.C. , Schmitz A., Reeder T.W. 2005. Partitioned Bayesian analyses, partition choice, and the phylogenetic relationships of scincid lizards. Syst. Biol. 54:373–390. 16012105 10.1080/10635150590946808

[CIT0007] Brown J.M. , Lemmon A.R. 2007. The importance of data partitioning and the utility of Bayes factors in Bayesian phylogenetics. Syst. Biol. 56:643–655. 17661232 10.1080/10635150701546249

[CIT0008] Burnham K.P. , Anderson D.R. 2002. Model selection and multi-model inference: a practical information-theoretic approach. Springer-Verlag, New York, NY.

[CIT0009] Cameron S.L. , Lo N., Bourguignon T., Svenson G.J., Evans T.A. 2012. A mitochondrial genome phylogeny of termites (Blattodea: Termitoidae): robust support for interfamilial relationships and molecular synapomorphies define major clades. Mol. Phylogenet. Evol. 65:163–173. 22683563 10.1016/j.ympev.2012.05.034

[CIT0010] Claeskens G. , Hjort N.L. 2008. Model selection and model averaging. Cambridge University Press, Cambridge.

[CIT0011] Crotty S.M. , Holland B.R. 2022. Comparing partitioned models to mixture models: Do information criteria apply? Syst. Biol. 71:1541–1548. 35041002 10.1093/sysbio/syac003 PMC9558833

[CIT0012] Crotty S. , Minh B.Q., Bean N.G., Holland B.R., Tuke J., Jermiin L.S., Haeseler A.V. 2020. GHOST: recovering historical signal from heterotachously evolved sequence alignments. Syst. Biol. 69:249–264. 31364711 10.1093/sysbio/syz051

[CIT0013] Darriba D. , Posada D. 2015. The impact of partitioning on phylogenomic accuracy. bioRxiv: 023978, preprint: not peer reviewed.

[CIT0014] Dziak J.J. , Coffman D.L., Robinson J., Lanza S.T., Li R., Jermiin L.S. 2020. Sensitivity and specificity of information criteria. Brief. Bioinform. 21:553–565. 30895308 10.1093/bib/bbz016 PMC7299313

[CIT0015] Felsenstein J. 2004. Inferring phylogenies. Sunderland, MA: Sinauer Associates.

[CIT0016] Gascuel O. 2005. Mathematics of evolution and phylogeny. OUP Oxford, New York.

[CIT0017] Hastie T. , Tibshirani R., Friedman J.H. 2009. The elements of statistical learning: data mining, inference, and prediction. 2nd ed. Springer Series in Statistics, Springer, New York.

[CIT0018] Ho S.Y. , Lanfear R. 2010. Improved characterisation of among-lineage rate variation in cetacean mitogenomes using codon-partitioned relaxed clocks. Mitochondrial DNA 21:138–146. 20795783 10.3109/19401736.2010.494727

[CIT0019] Holder M.T. , Lewis P.O., Swofford D.L. 2010. The Akaike information criterion will not choose the no common mechanism model. Syst. Biol. 59:477–485. 20547783 10.1093/sysbio/syq028

[CIT0020] Jayaswal V. , Wong T.K., Robinson J., Poladian L., Jermiin L.S. 2014. Mixture models of nucleotide sequence evolution that account for heterogeneity in the substitution process across sites and across lineages. Syst. Biol. 63:726–742. 24927722 10.1093/sysbio/syu036

[CIT0021] Jhwueng D. , Huzurbazar S., O’Meara B.C., Liu L. 2014. Investigating the performance of AIC in selecting phylogenetic models. Stat. Appl. Genet. Mol. Biol. 13:459–475. 24867284 10.1515/sagmb-2013-0048

[CIT0022] Kainer D. , Lanfear R. 2015. The effects of partitioning on phylogenetic inference. Mol. Biol. Evol. 32:1611–1627. 25660373 10.1093/molbev/msv026

[CIT0023] Kim T.L. , Sy V.L. 2020. mPartition: a model-based method for partitioning alignments. J. Mol. Evol. 88:641–652. 32864711 10.1007/s00239-020-09963-z

[CIT0024] Kuhner M.K. , Felsenstein J. 1994. A simulation comparison of phylogeny algorithms under equal and unequal evolutionary rates. Mol. Biol. Evol. 11:459–468. 8015439 10.1093/oxfordjournals.molbev.a040126

[CIT0025] Kullback S. , Leibler R.A. 1951. On information and sufficiency. Ann. Math. Stat. 22:79–86.

[CIT0026] Lanfear R. , Calcott B., Ho S.Y., Guindon S. 2012. PartitionFinder: combined selection of partitioning schemes and substitution models for phylogenetic analyses. Mol. Biol. Evol. 29:1695–1701. 22319168 10.1093/molbev/mss020

[CIT0027] Lanfear R. , Calcott B., Kainer D., Mayer C., Stamatakis A. 2014. Selecting optimal partitioning schemes for phylogenomic datasets. BMC Evol. Biol. 14:821–814. 10.1186/1471-2148-14-82 PMC4012149 24742000

[CIT0028] Lanfear R. , Frandsen P.B., Wright A.M., Senfeld T., Calcott B. 2017. PartitionFinder 2: new methods for selecting partitioned models of evolution for molecular and morphological phylogenetic analyses. Mol. Biol. Evol. 34:772–773. 28013191 10.1093/molbev/msw260

[CIT0029] Lartillot N. , Philippe H. 2004. A Bayesian mixture model for across-site heterogeneities in the amino-acid replacement process. Mol. Biol. Evol. 21:1095–1109. 15014145 10.1093/molbev/msh112

[CIT0030] Le S.Q. , Lartillot N., Gascuel O. 2008. Phylogenetic mixture models for proteins. Philos. Trans. R. Soc. B Biol. Sci. 363:3965–3976. 10.1098/rstb.2008.0180 PMC2607422 18852096

[CIT0031] Leavitt J.R. , Hiatt K.D., Whiting M.F., Song H. 2013. Searching for the optimal data partitioning strategy in mitochondrial phylogenomics: a phylogeny of Acridoidea (Insecta: Orthoptera: Caelifera) as a case study. Mol. Phylogenet. Evol. 67:494–508. 23454468 10.1016/j.ympev.2013.02.019

[CIT0032] Li C. , Lu G., Orti G. 2008. Optimal data partitioning and a test case for ray-finned fishes (Actinopterygii) based on ten nuclear loci. Syst. Biol. 57:519–539. 18622808 10.1080/10635150802206883

[CIT0033] Lopez P. , Casane D., Philippe H. 2002. Heterotachy, an important process of protein evolution. Mol. Biol. Evol. 19:1–7. 11752184 10.1093/oxfordjournals.molbev.a003973

[CIT0034] Luo A. , Qiao H., Zhang Y., Shi W., Ho S.Y., Xu W., Zhang A., Zhu C. 2010. Performance of criteria for selecting evolutionary models in phylogenetics: a comprehensive study based on simulated datasets. BMC Evol. Biol. 10:1–13. 20696057 10.1186/1471-2148-10-242 PMC2925852

[CIT0035] McGuire J.A. , Witt C.C., Altshuler D.L., Remsen J.V. 2007. Phylogenetic systematics and biogeography of hummingbirds: Bayesian and maximum likelihood analyses of partitioned data and selection of an appropriate partitioning strategy. Syst. Biol. 56:837–856. 17934998 10.1080/10635150701656360

[CIT0036] Minh B.Q. , Schmidt H.A., Chernomor O., Schrempf D., Woodhams M.D., Von Haeseler A., Lanfear R. 2020. IQ-TREE 2: new models and efficient methods for phylogenetic inference in the genomic era. Mol. Biol. Evol. 37:24611530–24612461. 10.1093/molbev/msaa015 PMC7182206 32011700

[CIT0037] Moody J. 1992. The effective number of parameters: an analysis of generalization and regularization in nonlinear learning systems. Neural Inf. Process. Syst. 4: 847–854.

[CIT0038] Neath A.A. , Cavanaugh J.E. 2012. The Bayesian information criterion: background, derivation, and applications. Wiley Interdiscip. Rev. Comput. Stat. 4:199–203.

[CIT0039] Ota R. , Waddell P.J., Hasegawa M., Shimodaira H., Kishino H. 2000. Appropriate likelihood ratio tests and marginal distributions for evolutionary tree models with constraints on parameters. Mol. Biol. Evol. 17:798–803. 10779540 10.1093/oxfordjournals.molbev.a026358

[CIT0040] Pagel M. , Meade A. 2004. A phylogenetic mixture model for detecting pattern-heterogeneity in gene sequence or character-state data. Syst. Biol. 53:571–581. 15371247 10.1080/10635150490468675

[CIT0041] Posada D. , Buckley T.R. 2004. Model selection and model averaging in phylogenetics: advantages of Akaike information criterion and Bayesian approaches over likelihood ratio tests. Syst. Biol. 53:793–808. 15545256 10.1080/10635150490522304

[CIT0042] Posada D. , Crandall K.A. 2001. Selecting the best-fit model of nucleotide substitution. Syst. Biol. 50:580–601. 12116655

[CIT0043] R Core Team. 2019. R: a language and environment for statistical computing. Vienna, Austria: R Foundation for Statistical Computing. Available from: URL https://www.R-project.org/.

[CIT0044] Rambaut A. , Grass N.C. 1997. Seq-Gen: an application for the Monte Carlo simulation of DNA sequence evolution along phylogenetic trees. Bioinformatics 13:235–238. 10.1093/bioinformatics/13.3.235 9183526

[CIT0045] Rao R. , Wu Y. 1989. A strongly consistent procedure for model selection in a regression problem. Biometrika 76:369–374.

[CIT0046] Rau A. , Maugis-Rabusseau C. 2018. Transformation and model choice for RNA-seq co-expression analysis. Brief. Bioinform. 19:425–436. 28065917 10.1093/bib/bbw128

[CIT0047] Rota J. , Wahlberg N. 2012. Exploration of data partitioning in an eight-gene data set: phylogeny of metalmark moths (Lepidoptera, Choreutidae). Zool. Scr. 41:536–546.

[CIT0048] Schliep K.P. 2011. phangorn: phylogenetic analysis in R. Bioinformatics 27:592–593. 21169378 10.1093/bioinformatics/btq706 PMC3035803

[CIT0049] Schwarz G. 1978. Estimating the dimension of a model. Ann. Stat. 6:461–464.

[CIT0050] Self S.G. , Liang K. 1987. Asymptotic properties of maximum likelihood estimators and likelihood ratio tests under nonstandard conditions. J. Am. Stat. Assoc. 82:605–610.

[CIT0051] Spiegelhalter D.J. , Best N.G., Carlin B.P., Van Der Linde A. 2002. Bayesian measures of model complexity and fit. J. R. Stat. Soc. Ser. B Statist. Methodol. 4:583–639.

[CIT0052] Sullivan J. , Joyce P. 2005. Model selection in phylogenetics. Annu. Rev. Ecol. Evol. Syst. 36:445–466.

[CIT0053] Susko E. , Roger A.J. 2020. On the use of information criteria for model selection in phylogenetics. Mol. Biol. Evol. 37:549–562. 31688943 10.1093/molbev/msz228

[CIT0054] Tagliacollo V.A. , Lanfear R. 2018. Estimating improved partitioning schemes for ultraconserved elements. Mol. Biol. Evol. 35:1798–1811. 29659989 10.1093/molbev/msy069 PMC5995204

[CIT0055] Whelan N.V. , Halanych K.M. 2017. Who let the CAT out of the bag? Accurately dealing with substitutional heterogeneity in phylogenomic analyses. Syst. Biol. 66:232–255. 27633354 10.1093/sysbio/syw084

[CIT0056] Zhou Y. , Rodrigue N., Lartillot N., Philippe H. 2007. Evaluation of the models handling heterotachy in phylogenetic inference. BMC Evol. Biol. 7:1–13. 17974035 10.1186/1471-2148-7-206 PMC2248194

